# Supplementary material for: Intrahousehold Food Intake Inequality by Family Roles and Age Groups
Source: Nutrients. 2023 Apr 28;15(9):2126. doi: 10.3390/nu15092126 (PMC10180906; doi:10.3390/nu15092126)
Supplement: Supplementary file 1 [file nutrients-15-02126-s001.zip › nutrients-2312610-supplementary.pdf]

Supplementary Materials

Estimation of food bundles and Poverty

The official methodology used in Bangladesh to estimate the poverty is based on the Cost of Basic Need (CBN) method. The CBN method calculates the cost of obtaining a consumption bundle considered to be adequate to satisfy the basic consumption needs. If a person cannot afford the cost of this bundle, then this person is considered poor (Bangladesh Bureau of Statistics, 2018). According to Isidoro (2005), a poverty threshold by CBN method is computed as follows:

1. Estimating the expenditure of the food basket as the food poverty line (fpl).
2. Estimating the expenditure of the non-food needs as the non-food poverty line (nfpl).
3. Adding both food and non-food poverty lines and obtaining the total poverty line (tpl).

Firstly, in order to estimating the food poverty line, data of the cost of basic food consumption is required. In Bangladesh, the food basket includes food items such as, rice, wheat, pulses, milk, oil, meat, fish, potatoes, other vegetables, sugar, fruits and others, as recommended by Ravallion and Sen (1996) and Alamgir (1974). After collecting the information about the food basket, then the price of food bundle is obtained by using per unit price of each food item. The food poverty line is calculated for each household by multiplying the estimated prices with the quantities in the food bundle. The food poverty line (fpl) can be written as follows:

$$\text{fpl} = \sum_{i=1}^{f} q_i p_i$$

where $q_1, \ldots q_f$ denote the quantity of each food item (i = 1, …, f) and $p_1, \ldots p_f$ relate to the unit price of each food item.

Secondly, the non-food basket by fully listing the non-food needs and calculating the price-weighted sum of the basket as the non-food poverty line (nfpl), similar to the food basket. In this study, the nonfood items include expenditures of fuel and lightning, transport and travel, clothing, health, housing, education, recreation and leisure followed by the guidelines of Bangladesh Bureau of Statistics.

In the next step, non-food expenditure of household is added to the food expenditure to get the total expenditure of a household, considering as total poverty line (tpl) and divided by the household size to have per capita expenditure per month. The definition of tpl as follows:

$$\text{tpl} = \text{fpl} + \text{nfpl}$$

The CBN approach is known as an official methodology for estimating poverty in Bangladesh, where a household under absolute poverty is one whose per capita expenditure is below the upper poverty line.

Reference

Alamgir, M. Some analysis of distribution of income, consumption, saving and poverty in Bangladesh. *Bangladesh Dev. Stud.* **1974**, *2*, 737–818.

Bangladesh Bureau of Statistics. District statistics. Technical report, Bangladesh Bureau of Statistics, 2018.

Isidoro, D. Country practices in compiling poverty statistics. *Handbook of Poverty Statistics*, United Nations Statistics Division, 2005.

Ravallion, M.; Sen, B. When method matters: Monitoring poverty in Bangladesh. *Econ. Dev. Cult. Change*. **1996**, *44*, 761–792.
